# Maimonides’ Appreciation for Medicine

**DOI:** 10.5041/RMMJ.10018

**Published:** 2011-01-31

**Authors:** Benjamin Gesundheit

**Affiliations:** Department of Jewish Philosophy, Hebrew University Jerusalem, Israel; and The International Center for Cell Therapy & Cancer Immunotherapy (CTCI), Weizmann Center, Tel Aviv, Israel

**Keywords:** Maimonides, motivation for medicine, Jewish medical ethics

## Abstract

Moses Maimonides, the illustrious medieval rabbi and philosopher, dedicated the last decade of his life primarily to medicine. His strong interest in medicine was an integral component of his religious-philosophical teachings and world view. In this paper various sources from his rabbinic writings are presented that explain Maimonides’ motivation regarding and deep appreciation for medicine: (A) The physician fulfills the basic biblical obligation to return lost objects to their owner, for with his knowledge and experience the physician can restore good health to his sick fellow human being; (B) medicine provides a unique opportunity to practice *imitatio dei*, as it reflects the religious duty to maintain a healthy life-style; (C) as an important natural science, medicine offers tools to recognize, love, and fear God. These three aspects address man’s relationship and obligation towards his fellow-man, himself and God. Biographical insights supported by additional sources from Maimonides’ writings are discussed.

## INTRODUCTION

Moses Maimonides, renowned Talmudist, philosopher, Jewish community leader, and physician, harmoniously integrated in his life outstanding achievements in various fields.^1^ Already in his early Rabbinic and philosophical writings, he demonstrates his high regard for the value of medicine and the natural sciences.^2^–^4^

This paper presents three sections (A, B, and C) from Maimonides’ rabbinical writings that deal with his motive for studying science and medicine. These three passages will be carefully analyzed, their sources in classical Jewish literature^5^–^7^ will be identified, and Maimonides’ contribution to their interpretation will be demonstrated. The contexts of these excerpts from Maimonides’ writings will be examined to see whether they shed light on Maimonides’ personality and thoughts. The relevant messages for contemporary physicians will be presented in the discussion.

## A. MEDICINE AS A FULFILLMENT OF THE BIBLICAL OBLIGATION TO RETURN LOST OBJECTS TO THEIR OWNER

Maimonides’ *Commentary to the Mishnah* (∼1168), written before he reached the age of thirty, already includes many of the insights found in his later Rabbinic and philosophical writings. Even though it was only during the last decade of his life that he was most active as a physician, Maimonides clearly indicates his interest and appreciation for science and medicine in his *Commentary to the Mishnah* written in a much earlier period of his life.^8^

Two passages in his *Commentary to the Mishnah* reflect two different aspects of medicine in Maimonides’ thought (A, B).

Inasmuch as according to Biblical sources “God is your healer”,^9^ the Talmud (fourth to fifth century CE) questions the ideological legitimacy of the practice of medicine; from another Biblical source it elaborates a special allowance to heal,^10^ which was in fact to be understood as a religious *duty* to heal, and this approach was accepted by most classical rabbinical authors.^11^,^12^ Maimonides did not accept this well known apologetic attitude of the Talmud towards medicine based on his philosophy of life (*Weltanschauung*), and he was the first Rabbinic author to understand medicine as a fundamental and a-priori religious duty anchored in a well known Biblical source and further supported by its Talmudic interpretation:

… because it is a religious duty, that is to say, a physician is obligated by law to heal the sick of Israel. This is included in what [the Sages] said in explanation of the verse, “And you shall restore it to him” (Deut. 22:2) – to include his body, that if he saw him lost and he can save him, he must save him with his body or with his money or with his knowledge…^13^

The Torah commands the return of lost objects to their owner: “You shall not see your brother’s ox or his sheep go astray, and hide yourself from them; you shall surely bring them back to your brother. And if your brother be not near to you, or if you know him not, then you shall bring it to your own house, and it shall be with you until your brother seeks after it, and you shall restore it to him. In like manner you shall do with his ass; and so shall you do with his garment; and with every lost thing of your brother’s, which he has lost, and you have found, shall you do likewise; you must not hide yourself”^14^ (compare Exodus 23:4). Since “every lost thing of your brother’s” is explicitly mentioned in this Biblical source, it is self-evident to the young Maimonides – with strong scientific and medical interests – that offering medical help is included in this fundamental Biblical duty. The physician’s knowledge and understanding of disease meets the Biblical definition of “and you have found” and, therefore, the physician is obligated to heal. Furthermore, acquiring scientific knowledge, which includes also medicine and natural sciences, is according to Maimonides in itself a religious duty in that it enhances a person’s recognition and love of God (see C below). The Arabic expression for “knowledge” in the quoted section from Maimonides’ *Commentary to the Mishnah* indicates the formal study of knowledge, and not just knowing something by accident. Later Rabbinic authors suggested that the study of medicine is a Biblical obligation so that one is capable of restoring to patients their lost health.^7^

Maimonides further supports his interpretation of the Biblical text with a well known Talmudic text. The Talmud extends the Biblical commandment to return lost objects to their owner to the case of a person who is lost, that the person who finds him is obligated to point him in the direction of his destination.^15^,^16^ Accordingly, a physician who is capable of diagnosing a sick person has the Biblical-Rabbinic obligation to offer him treatment. By doing so, he “returns” to the patient his lost item, i.e. his health and well-being, and fulfills thereby a religious commandment.^17^

Inasmuch as the verse speaks of the lost objects of “your brother”, Maimonides applies this law here to the treatment of Jewish patients. In his *Mishneh Torah*, however, Maimonides writes that where there is the possibility of the desecration of God’s name and as part of the “ways of peace”, one must also return the lost objects belonging to a Gentile,^18^ and so presumably he must also offer them medical treatment. And indeed Maimonides treated Jewish and non-Jewish patients alike, as he reports in one of his letters: “I find the antechambers filled with people, both Jews and Gentiles, nobles and common people, judges and policemen, friends and foes – a mixed multitude, who await the time of my return.”^19^

According to Maimonides, there is a religious duty to offer medical help to any sick person in need – Jew and non-Jew alike – based on the classical Biblical-Talmudic obligation to return lost objects to their owner.

By the same token, providing halakhic and social support to the Jewish community in Yemen suffering from severe persecutions was for Maimonides a medical service and obligation. In his famous letter to this community in need (*Iggeret Teiman = Epistle to Yemen*), Maimonides compares their poor political-religious condition to a disease and metaphorically considers his halakhic guidance to resolve their unbearable situation as a therapy. Maimonides obviously understands medicine in the broader sense of offering help to people in need:

… Realizing this amazing matter that hurts the eyes, I undertook to gather pharmaceutics and roots from the books of the ancients, of which I intend to prepare medicine and salve helpful for this sickness, and I heal it with the help of God…^20^

Maimonides defines medicine as a basic religious obligation based on his new interpretation of a well known Biblical verse. Medicine is highly respected in Jewish tradition, but according to Maimonides’ understanding medical practice and research is directly rooted in the most classical of Jewish sources.

## B. MEDICINE AS *IMITATIO DEI* AND SELF-GUIDANCE TO A HEALTHY LIFE-STYLE

One of Maimonides’ fundamental philosophical principles is man’s duty to adhere to the golden mean, meaning the felicitous middle between the extremes of excess and deficiency.^21^ Maimonides applies this philosophical principle both to religious-spiritual qualities and medical guidelines for a healthy life-style. Man must take the proper steps to maintain his good health and well-being. According to this approach, not only is medicine not antithetical to religion and belief in God’s providence, as it is perceived in other religious traditions;^22^ but, on the contrary, man is obligated to imitate God and act as His agent, as it were, by improving His world and treating those of His creatures who are in need.

It is quite common for one concept to appear in several places in Maimonides’ writings, with different aspects of the same concept being presented and elaborated upon in each place.

… On the basis of this reasoning, the art of medicine is given a very large role with respect to the virtues, the knowledge of God, and attaining true happiness. To study it diligently is among the greatest acts of worship. It is, then, not like weaving and carpentry, for it enables us to perform our actions so that they become human actions, leading to the virtues and the truths…^23^

In his *Mishneh Torah* (1180s), Maimonides further elaborates upon his concept of the golden mean: A person should constantly train himself to walk in God’s ways and acquire His qualities (*imitatio Dei*); this is the heritage which the Patriarch Abraham passed down to his descendants.^24^ Finding the golden mean is not always an easy task, and therefore, in order to achieve a healthy life-style,^25^–^27^ one might need further guidance. Maimonides advises those who are ill to consult with the wise, whom he refers to in this context as “healers of the soul”,^28^ a unique Maimonidean term reflecting his awareness of psychosomatics.^29^ He further develops the medical rules according to which a person must train himself to live,^30^ as physical health is a precondition for a healthy mind, which is a requisite for recognizing God.^31^ This statement by Maimonides reminds us of the well known Latin proverb: “*Mens sana in corpore sano*” (“A healthy mind in a healthy body”, or “A sound mind in a sound body”), but Maimonides anchors this concept within Rabbinic sources.

While Maimonides presents this religious concept as a Biblical commandment in his *Commentary to the Mishnah* and in his *Mishneh Torah*, he further elaborates upon the medical details of self-guidance in his medical writings, especially in his *Guide to Good Health* (*Regimen Sanitatis*) (1198).^32^ In the introduction to his philosophical *magnum opus*, the *Guide for the Perplexed* (1190s), as well, Maimonides encourages his philosophically perplexed reader to find the right path in order to be “healed”:

... To sum up: I am the man who when the concern pressed him and his way was straitened and he could find no other device by which to teach a demonstrated truth other than by giving satisfaction to a single virtuous man while displeasing ten thousand

ignoramuses – I am he who prefers to address that single man by himself, and I do not heed the blame of those many creatures. For I claim to liberate that virtuous one from that into which he has sunk and I shall guide him in his perplexity until he becomes perfect and is healed…^33^

According to these sources, a person is obligated to maintain his own physical and spiritual health and well-being. This obligation is based on the Biblical command to find and follow the golden mean in order to protect one’s health.

While the previous Biblical-Talmudic source (A) addressed the obligation to assist a sick person in need, this source (B) sees a healthy life-style, which includes proper diet and hygiene, as a set of preventive measures to avoid sicknesses, which is a most important goal according to Maimonides’ thinking.^34^–^36^ A healthy life-style is not only advisable; it is part of the religious duty to imitate God, and, therefore, it is a religious obligation.

## C. MEDICINEAND NATURAL SCIENCES – A TOOL TO RECOGNIZE, LOVE, AND FEAR GOD

According to the two sources discussed above, medicine is an important practical means for helping one’s fellow-man in need (A), as well as an instrument for preserving one’s own physical and spiritual well-being (B). There is, however, another important reason to study medicine: The study of natural sciences is the ideal way to recognize God and consecutively to love and fear Him:

... This God, honored and revered, it is our duty to love and fear; as it is said: “You shall love the Lord, your God” (Deut. 6:5), and it is further said: “You shall fear the Lord, your God” (Deut. 6:13). And what is the way that will lead to the love of Him and the fear of Him? When a person contemplates His great and wondrous works and creatures and from them obtains a glimpse of His wisdom which is incomparable and infinite, he will immediately love Him, praise Him, glorify Him, and long with an exceeding longing to know His great name; even as David said: “My soul thirsts for God, for the living God” (Ps. 42:3). And when he ponders these matters, he will recoil affrighted, and realize that he is a small creature, lowly and obscure, endowed with slight and slender intelligence, standing in the presence of Him who is perfect in knowledge. And so David said: “When I consider Your heavens, the work of Your fingers – what is man that You are mindful of him?” (Ps. 8:4–5). In harmony with these sentiments, I shall explain some large, general aspects of the Works of the Sovereign of the Universe, that they may serve the intelligent individual as a door to the love of God, even as our sages have remarked in connection with the theme of the love of God, “Observe the Universe and hence, you will realize Him who spoke and the world was...”^37^

Studying the natural sciences including medicine is a fulfillment of the religious duty to recognize, love, and fear God, on the one hand, and to develop the appropriate attitude and modesty for human beings, on the other. Maimonides supports his position with Biblical sources (Ps. 42:3; 8:4). In one of his letters he calls the natural sciences the “maidservants of Jewish studies”, as they are crucial for the proper understanding of the world.^38^

Interestingly, Maimonides elaborates upon the commandment to love and fear God in his *Sefer ha-Mitzvot*, the *Book of Commandments* (1172), which summarizes all 613 Biblical commandments. But whereas in his *Mishneh Torah* he recommends the observation and study of nature as a means to know, love, and fear God, in his *Sefer ha-Mitzvot* he recommends the study of Scripture to achieve these objectives. This apparent contradiction can easily be explained based on the different contexts: In his strictly rabbinic *Sefer ha-Mitzvot*, Maimonides remains focused upon the religious commandments, and therefore he bases the love of God on the study of those commandments; in his halakhic-philosophical work, *Mishneh Torah*, philosophical and scientific aspects play a central role,^39^ and, therefore, the study of the natural sciences provides an additional path to recognize, love, and fear God.^40^ Indeed, in his philosophical *Guide for the Perplexed* he further elaborates upon this position (“We have already explained in Mishneh Torah that this love becomes valid only through the apprehension of the whole of being as it is and through the consideration of His wisdom as it is manifested in it.”),^41^ which he also recommends in practice in his Responsa.^42^ Therefore, Maimonides points out already in his *Commentary to the Mishnah* that in order to permit the violation of religious obligations for medical reasons, medicine has to be based on strong rational, scientific, and clinical experience.^43^

## DISCUSSION

Maimonides integrated medicine and science into his own *Weltanschauung* which is deeply anchored in the classical Jewish sources. The three different aspects presented in this essay illustrate Maimonides’ deep appreciation for medicine. They reflect man’s three fundamental relationships – to his fellow-man, to himself, and to God:

1. Medicine as a fulfillment of the Biblical obligation to return lost objects to their ownerdescribes the physician’s obligation to help his fellow-man in acute need.
  Medicine as *imitatio Dei* and self-guidance to a healthy life-style explains each individual’s obligation towards himself to follow the golden mean and thus advance his physical health.
  Medicine and natural sciences are tools to recognize, love, and fear God, creating a religious attitude for a religious scientist to experience God through the natural sciences.

While the classical medical oaths, such as that of Hippocrates,^44^ Assaph (sixth century, Israel),^45^ Rabbi Yaakov Zahalon (seventeenth century, Rome),^46^,^47^ and others,^48^ reflect the belief system of their authors as individual physicians and historical representatives of their cultural environments, the above-mentioned sources from Maimonides’ writings on medicine are directly rooted in the classical sources of Jewish tradition and reflect basic religious duties and principles. For his monumental systematization of Jewish literature Maimonides was praised as “architect of Jewish studies”;^49^ for his ideological integration of medicine into Jewish tradition, known historically from many other famous rabbi-physicians,^50^ Maimonides might further deserve the title “Rabbi of Medicine”.^51^

While the schematic presentation of these three different aspects is by its nature theoretical, Maimonides’ life demonstrates his practical appreciation for medicine, including his love for human beings, especially those in need.^52^ For Maimonides, providing help and joy to people in need is not only a technical fulfillment of a formal religious duty; this action brings happiness to the benefactor and provides him with the opportunity to imitate God. The fusion of these three aspects is demonstrated in one of Maimonides’ halakhic rulings:

… For no joy is greater or more glorious than the joy of gladdening the hearts of the poor, the orphans, the widows, and the strangers. Indeed, he who causes the hearts of these unfortunates to rejoice emulates the Divine Presence, of whom Scripture says: “To revive the spirit of the humble, and to revive the heart of the contrite ones” (Is. 57:15)...”.^53^

This rather technical context regarding laws governing a Jewish festival inspires Maimonides to elaborate on the related ethical and philosophical aspects of Jewish tradition,^54^,^55^ reflecting his enthusiasm to help people in need. Once again he uses a Biblical source from Isaiah 57:15 to prove his point that by supporting needy people one imitates God in an ideal way.^56^

**Figure 1:**
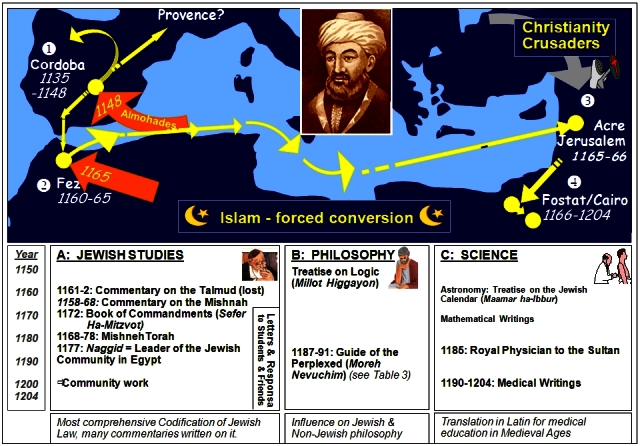
Moses Maimonides (1135–1204): His life and writings. (Modified from Gesundheit B, Hadad E,^1^ reprinted withpermission).

At the end of his *Guide for the Perplexed* (1190s), Maimonides describes the highest level of religious and existential action based on Biblical sources: “I am the Lord who exercises mercy, justice, and righteousness on earth; for in these things I delight, said the Lord.”^57^ Medicine is indeed the ideal instrument through which to fulfill these religious and philosophical values in practice. Upon examination of Maimonides’ biography and writings (Figure 1),^58^ it is evident that during his later years Maimonides translated these philosophical principles into actual practice and focused his productive life primarily upon medical practice and research, as is documented in his medical writings.^59^ In a letter written during this period, Maimonides states that he has no time for the study of the Torah^60^ because his time is fully dedicated to medical practice: “ … I go forth to attend to my patients, and write prescriptions and directions for their various ailments. Patients go in and out until nightfall, and sometimes even … until two hours or more into the night. I converse with them and prescribe for them even while lying down from sheer fatigue; and when night falls, I am so exhausted that I can hardly speak”.^19^

In his medical writings he demonstrates tremendous respect in the care for his patients. For example, with one of his Islamic patients, he shares and discusses the philosophical values of Jewish tradition and also those of the patient’s own religion and belief system.^61^ Though not written by Maimonides himself,^62^ the famous *Prayer for the Physician* attributed to him concisely articulates the medical calling and underlying ethical values of the physician.

Maimonides’ appreciation of the study of medicine is reflected also in other places in his writings: Maimonides requires that judges sitting on a Jewish court should have basic knowledge in medicine,^63^ a feature not mentioned in the Talmudic source listing the skills required of judges.^64^ In the introduction to his commentary on Hippocrates’ writings, Maimonides suggests that these medical topics be taught also to non-physicians as part of their general education, since medical knowledge is useful and important for everyone.^65^ It is not surprising then that Maimonides expected his own students to acquire knowledge in the medical sciences: he guides his famous student Rabbi Joseph ben Judah ibn Aknin (*c.* 1150–1220) to support himself through business dealings and to study medicine along with the classical Jewish texts.^60^ Indeed, this very same student clarifies the religious meaning of medical science and practice in one of his rabbinic writings.^66^

Owing to his unique integration of classical Jewish values and philosophical virtues into medical practice, Maimonides – after more than 800 years – remains an outstanding role model for contemporary physicians.
